# Bispecific Antibodies in Prostate Cancer Therapy: Current Status and Perspectives

**DOI:** 10.3390/cancers13030549

**Published:** 2021-02-01

**Authors:** Jonas S. Heitmann, Martin Pfluegler, Gundram Jung, Helmut R. Salih

**Affiliations:** 1 Clinical Collaboration Unit Translational Immunology, German Cancer Consortium (DKTK), Department of Internal Medicine, University Hospital Tübingen, 72076 Tübingen, Germany; jonas.heitmann@med.uni-tuebingen.de (J.S.H.); m.pfluegler@dkfz-heidelberg.de (M.P.); 2 DFG Cluster of Excellence 2180 “Image-Guided and Functional Instructed Tumor Therapy” (IFIT), University of Tübingen, 72076 Tübingen, Germany; gundram.jung@uni-tuebingen.de; 3 Department of Immunology, Interfaculty Institute for Cell Biology, University of Tübingen, 72076 Tübingen, Germany; 4 German Cancer Consortium (DKTK), DKFZ Partner Site Tübingen, 72076 Tübingen, Germany

**Keywords:** bispecific antibody, prostate cancer, CRPC

## Abstract

**Simple Summary:**

Despite recent advances in treatment, metastasized prostate carcinoma (PC) still has a poor prognosis. Immunotherapy has revolutionized the landscape of cancer therapy, but a breakthrough for PC is missing. The success of immunotherapy in cancers is mostly due to recent strategies to mobilize T cells comprising immune checkpoint inhibition, CAR-T cells and bispecific antibodies (bsAbs). After introducing present approaches and immunotherapy in PC in general, we here review the current clinical development of bsAbs in PC treatment.

**Abstract:**

Prostate carcinoma (PC) is the second most common cancer in men. When the disease becomes unresponsive to androgen deprivation therapy, the remaining treatment options are of limited benefit. Despite intense efforts, none of the T cell-based immunotherapeutic strategies that meanwhile have become a cornerstone for treatment of other malignancies is established in PC. This refers to immune checkpoint inhibition (CI), which generally reinforces T cell immunity as well as chimeric antigen receptor T (CAR-T) cells and bispecific antibodies (bsAbs) that stimulate the T cell receptor/CD3-complex and mobilize T cells in a targeted manner. In general, compared to CAR-T cells, bsAb would have the advantage of being an “off the shelf” reagent associated with less preparative effort, but at present, despite enormous efforts, neither CAR-T cells nor bsAbs are successful in solid tumors. Here, we focus on the various bispecific constructs that are presently in development for treatment of PC, and discuss underlying concepts and the state of clinical evaluation as well as future perspectives.

## 1. Introduction

Prostate cancer (PC) is the second most common cancer in men worldwide, with 1,276,106 registered cases and 358,989 deaths in 2018 [1]. Androgen deprivation therapy continues to be the first-line therapy, but in many cases the disease is or becomes unresponsive to this treatment modality. Multiple drugs have been evaluated for this situation, but all are of limited success: Abiraterone and enzalutamide act on the androgen axis and slow down disease progression and improve overall survival (OS) to a moderate extent [2,3,4]. Treatment of patients with metastatic castration-resistant prostate cancer (CRPC) with the cytostatic drugs docetaxel and cabazitaxel results in a median OS benefit of up to 19.2 months [5,6].

At least in certain cancer indications, immunotherapy has in the recent years revolutionized the landscape of oncological treatment [7]. This particularly holds true for strategies recruiting T cells, the central components of the adaptive immune system. Physiologically, two different kinds of signals regulate T cell activation and thus specific immunity: “signal 1” is mediated by the antigen-specific T cell receptor/CD3 complex (TCR/CD3) that recognizes peptides bound to MHC molecules. Additional “second signals” via costimulatory and/or coinhibitory (“immune checkpoint”) receptors then determine whether a profound and long-lasting immune response is induced or not [8]. The receptors mediating these important effects have been characterized in the last three decades and enabled the development of effective T cell-based cancer treatment strategies: immune checkpoint inhibition (CI) prevents transduction of inhibitory signals via PD-1 and CTLA-4. The effect is illustrated by the picture of “releasing the brake” of antitumor immunity, which reinforces T cell reactivity in an undirected manner. Particularly in melanoma and lung cancer with metastatic disease, CI can induce long-lasting remissions even in patients with high tumor burden [9], but durable responses are so far achieved in a minor subset of patients only [10,11,12,13,14,15,16], and treatment is associated with considerable side effects due to the induction of autoimmune reactions. In contrast to CI, bispecific antibodies (bsAbs), which stimulate TCR/CD3 with their effector part after binding their target antigen on tumor cells, as well as the closely related chimeric antigen receptor T (CAR-T) cells, which—as an oversimplification—can be considered genetically modified T cells that carry an integrated bsAb (CD3 signaling unit anchored in the T cell), both aim for inducing target antigen-dependent and thus more directed antitumor immunity. Due to the crucial role of T cells in tumor immunosurveillance, in the past a lot of effort was made to define peptide motifs within tumor antigens which enable therapeutic vaccination strategies. Such approaches to induce tumor-reactive T cells have yielded promising results in terms of immunogenicity and first evidence of clinical efficacy [17,18]. A related approach is to utilize antigen-presenting cells (APCs) which physiologically regulate T cell responses [19,20]. As of today, the only clinically available immunotherapy using APC is approved for metastatic PC: sipuleucel-T consists of autologous APC incubated with a recombinant fusion protein (PA2024) composed of human prostatic acid phosphatase as tumor antigen linked to granulocyte–macrophage colony-stimulating factor. It prolonged OS among patients with metastatic CRPC in two phase III trials [21,22], but plays no major role in daily clinical routine.

With regard to the aforementioned T cell-based strategies, CI has so far not yielded convincing efficacy (for an excellent review see Kim et al. [23]). Approaches using CAR-T cells and bsAbs in PC are frequently directed to the same target antigens and also share many other similarities, but bsAbs provide the advantage of being a standardized “of the shelf reagent” with pharmacokinetically controllable activity. In this review, after briefly summarizing the current state of development of CI and CAR-T cells in PC, we discuss the various bsAb constructs that are presently in development in PC with a focus on compounds undergoing clinical evaluation in CRPC.

## 2. Checkpoint Inhibition in Prostate Cancer

Numerous clinical trials in patients with CRPC were conducted using the different compounds presently available for CI, this is, monoclonal antibodies directed to CTLA-4, PD-1, and PD-L1. So far, results were not satisfactory and demonstrated only limited survival benefit [10,11,12,13,14,15,16]. Thus, no CI strategy is so far available for treatment of PC patients, with the exception of the general FDA approval of pembrolizumab for malignancies with mismatch repair deficiency or a microsatellite instability-high status [24,25]. Notably, a phase I trial evaluating the combination of sipuleucel-T and ipilumumab in patients with CRPC reported enhanced immunological responses [26]. Multiple clinical trials (about 30 registered on clinicaltrials.gov, January 2021) evaluating combinatorial regimens comprising of different CI antibodies or their combination with other treatment modalities, or enrolling specific subgroups of PC patients, are presently ongoing and described in detail in the aforementioned excellent recent review by Kim and coworkers [23].

## 3. CAR-T Cells in Prostate Cancer

Compared to bsAbs, CAR-T cells presently receive far greater interest in the oncological community (156 versus 1174 ongoing clinical studies, clinicaltrials.gov, December 2020) [27]). Nevertheless, the disadvantages of CAR-T cells, like the time-consuming and costly individualized manufacturing process as well as the hardly controllable and persisting activity after application, in our view constitute drawbacks of this treatment modality [28] and argue in favor of bsAbs.

As of today, data from two phase I clinical trials evaluating CAR-T cells in PC are available, which both used the prostate-specific membrane antigen (PSMA) as target. PSMA is a type II transmembrane glycoprotein rather specifically expressed in PC cells [29] and will be described in greater detail below. Junghans et al. treated five metastatic CRPC patients with CAR-T cells, of which three achieved the 20% engraftment goal [30]. Two of these patients exhibited a PSA response, and neutropenic fever was observed in 5/5 patients whereas, quite surprisingly, a cytokine release syndrome (CRS) was not observed [30]. In another trial [31], two of the seven patients with metastatic CRPC treated with PSMA-CAR-T cells showed a response, and further evidence for potential clinical activity was deduced from one patient experiencing a long-term response over 16 months [31]. In an ongoing trial, CAR-T cells engineered to be insensitive to the immunosuppressant cytokine TGF-β are being evaluated, but results are as of yet not available [32,33].

## 4. BsAbs in Prostate Cancer

### 4.1. Background

Humanized monoclonal antibodies (mAbs) like rituximab and trastuzumab, which are employed for treatment of B cell lymphoma and Her2/neu-positive breast cancer, respectively, have considerably improved therapeutic options for these diseases and are nowadays well established in cancer treatment. Central for the therapeutic activity of mAbs is their ability to stimulate Fc-receptor (FcR)-bearing immune effector cells, which results in lysis of target cells mainly by NK cells [34].

**Figure 1:**
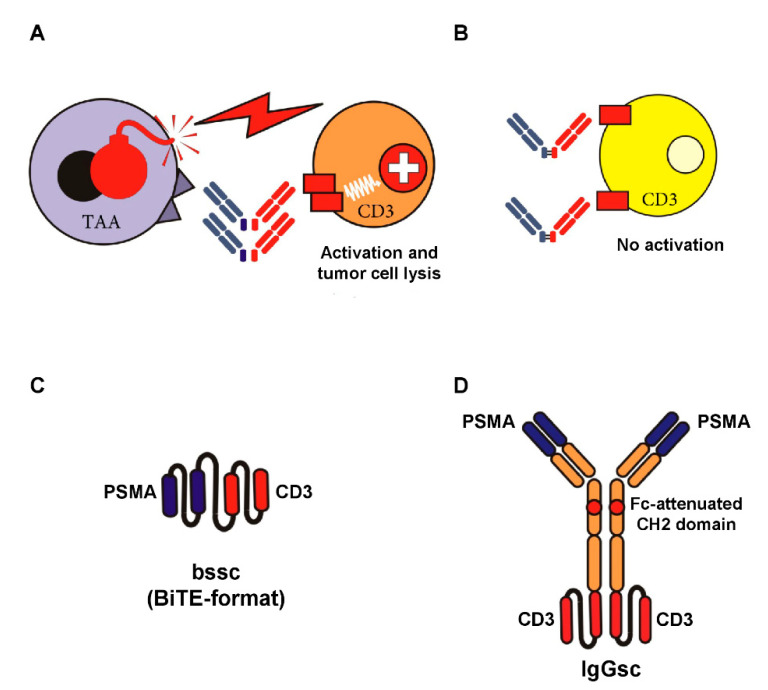
Target-restricted T cell activation with Fc-depleted bsAbs and examples of Fc-attenuated bispecific formats. BsAbs equipped with a target-specificity directed to a tumor-associated antigen (TAA) and an effector-specificity targeting an activating receptor on T cells, such as CD3, are capable of potently recruiting T cells against tumor cells (**A**). To avoid “off-target” activation in the absence of tumor cells (**B**), the selected target antigen should not be expressed on healthy cells, and binding of antibody Fc-parts to Fc-receptor-carrying cells has to be prevented. The latter can be achieved by recombinant DNA-technology using e.g., single-chain antibody fragments (**C**) or attenuation of the CH2 domain (IgG-based format like in CC-1, our bispecific PSMAxCD3 antibody in IgGsc format) depicted in (**D**).

An alternative approach is to aim for antibody-mediated stimulation of T cells with their—compared to NK cells—higher effector potential using bsAbs [35], which recognize a TAA with their target arm and, with their effector arm, stimulate the TCR/CD3 complex (see Figure 1A,B). Multiple formats of bsAbs have meanwhile been developed, and the field is growing rapidly. Accordingly, we focus this review to formats emerging to clinical application in CRPC; a general overview on bsAb formats that are presently in development is provided in an excellent review by Brinkmann and Kontermann [36]. The only clinically approved reagent for cancer therapy in the class of bsAbs is the CD19xCD3 bsAb Blinatumomab for treatment of B-ALL [37]. So far, sustained therapeutic success of this and many other bsAbs presently undergoing clinical evaluation is forestalled by the unspecific activation of the T cell system, resulting in a potentially life threatening CRS. This in turn limits application of sufficient dosing to achieve optimal antitumor efficacy. “On-target off-tumor” activation due to expression of the selected target antigens on normal human tissue significantly contributes to this undesired phenomenon. To prevent this situation, the selected target antigen should ideally not be expressed on healthy cells. Moreover, binding of bsAbs to Fc-receptor-carrying cells has to be prevented (see Figure 1C,D).

Additional problems of bsAbs constructed in “small formats” like the prototypical BiTE are their low serum half-life and aggregation tendency [38,39,40], which result in cumbersome continuous infusion protocols and potentially also “off-target” activation of T cells, respectively. Various strategies to overcome these limitations are being developed and comprise evaluation of alternative application forms (e.g., subcutaneous rather than intravenous) and development of further optimized bsAb constructs, respectively.

As of today, the effector arm of most bsAbs for cancer therapy, particularly in all compounds that presently are undergoing clinical evaluation, is directed to TCR/CD3 on T cells. However, in addition to the first signal via the TCR/CD3 complex, T cells require a second “costimulatory” signal for maximal and sustained activation [41]. In line, it was not before costimulatory signaling domains derived from CD28 and/or 4-1BB were included (in addition to CD3 derived motifs) that impressive therapeutic activity of CAR-T cells was achieved [42]. In the bsAb field, costimulation has so far received less attention, although bispecific CD28 antibodies were already preclinically and also clinically characterized many years ago [43,44]. In subsequent years, technical problems (production and characterization), but also the deleterious cytokine release observed after application of a “superagonistic” CD28 antibody, meanwhile known as the “Tegenero incident” [45,46], hampered the further clinical development of such bispecific costimulators (BiCos). However, very recently utilization of CD28 targeting BiCos is gaining renewed interest, as discussed in greater detail below.

### 4.2. Target Antigens for bsAbs in PC

#### 4.2.1. PSMA

**Figure 2:**
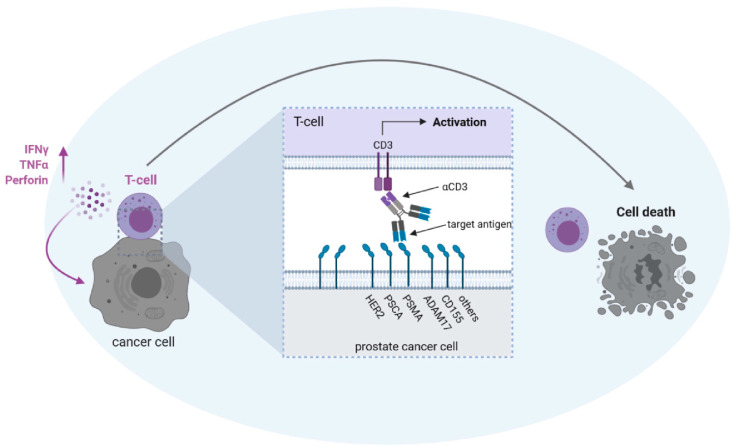
Possible antigen targets for T cell activation with bsAbs in prostate cancer. BsAbs equipped with a target-specificity directed to a tumor-associated antigen (HER2/neu = HER2; prostate stem cell antigen = PSCA; prostate-specific membrane antigen = PSMA; ADAM metallopeptidase domain 17 = ADAM17; poliovirus receptor = CD155) and an effector-specificity targeting an activating receptor on T cells, such as CD3, are capable of potently recruiting T cells against tumor cells. To avoid “off-target” activation in the absence of tumor cells, the selected target antigen should not be expressed on healthy cells and binding of antibody Fc-parts to Fc-receptor-carrying cells has to be prevented. The latter can be achieved either by depletion of the Fc-part or by modification of the Fc-part by recombinant DNA-technology (see also Figure 1).

The most prominent and widely used tumor-associated antigen (TAA) in PC is PSMA (Figure 2).

PSMA is expressed rather specifically on healthy prostate and PC cells, but notably also on the neovasculature of various different solid tumors, including PC [47,48,49,50]. In our view, the vascular expression is of particular importance: In the case of solid tumors, the therapeutic activity of bsAbs (and also CAR-T cells) so far does not match that achieved in hematopoietic malignancies, for the most part due to an insufficient influx of T cells to the tumor site. To overcome this limitation, an optimal target antigen should be expressed not only on tumor cells, but also on tumor vessels, allowing for “dual targeting” of both structures, which then may allow sufficient influx of immune cells via damaged endothelium and subsequent tumor cell destruction. This view is supported by reports demonstrating that T cells fail to eradicate established tumors unless a proinflammatory microenvironment is present which facilitates T cell extravasation [51]. The concept of dual antitumor action is further supported by findings that antibody drug conjugates (ADCs) directed to the target antigen CD276/B7-H3, which is expressed on both tumor cells and tumor vasculature, only effectively eradicate established tumors in mice when armed with a drug acting against tumor cells and simultaneously also the tumor vasculature [52].

Altogether, PSMA is the best established TAA in PC, which is also documented by its clinical utilization for imaging using radioactive PSMA tracers like ^68^Ga- and ^18^F-labeled compounds [53,54,55]. Combined with CT or MRT, at least in the metastatic situation, PSMA-PET has become part of routine care for CRPC patients. This was expanded by the development of the monoclonal PSMA antibody J591 bound, e.g., to ^177^Lutetium, which opened up the field of PSMA theranostics [47,56]. Accordingly, in the last decade, several studies reported on this and other PSMA targeting radiolabeled agents that showed clinical activity in CRPC [57,58,59,60]. In line, a plethora of preclinically characterized therapeutic constructs and almost all bsAbs that underwent clinical evaluation in PC are directed to PSMA [61,62,63,64].

#### 4.2.2. Other Targets

Several other TAAs are being evaluated as targets of bsAbs in PC (Figure 2):

The GPI-anchored cell surface protein PSCA (prostate stem cell antigen) is overexpressed in advanced PC [65], with a prevalence of about 90% in primary PC as well as bone, lymph node, and liver metastases [66,67,68]. In addition, PSCA is also upregulated in bladder and pancreatic cancers [69,70]. As of now, besides being used as target for vaccination strategies [71], one study evaluated single-chain bsAb targeting CD3 using PSCA as TAA in preclinical analyses [72].

CD155 is highly expressed on various different tumor types including PC, where it is reportedly associated with metastasis, but expression has been described for a variety of healthy cell types [73,74,75]. A CD155xCD3 bsAb was reported to stimulate the ability of ex vivo activated T lymphocytes to kill PC cells in vitro [76].

Her2/neu is well known as therapeutic target in breast cancer, but also considered as potential target in PC, where it is expressed on the tumors of up to 70% of patients [77,78]. In one clinical trial, activated T cells loaded with Her2xCD3 bsAbs were applied twice weekly for four weeks. In three out of seven patients with metastatic CRPC, a transient decrease of PSA was observed, and further evidence for potential clinical activity was deduced from one patient experiencing a partial response within 6 months of completing therapy. Grade 3 toxicity (chills) was reported for the majority of patients (5/7) [79].

ADAM17 (A disintegrin and metalloproteinase 17) is a membrane-bound protease that cleaves various cell surface proteins, including cytokines and cytokine receptors, and is highly expressed on tumor cells [80,81,82,83]. Yamamoto et al. described the generation of an ADAM17xCD3 bsAb in the BiTE format and conducted preclinical studies with regard to antigen selectivity and efficacy in killing PC tumor cells expressing ADAM17 in the presences of T cells [84].

## 5. Toxicity Considerations

As stated above, sustained therapeutic success of presently available bsAbs is so far forestalled by severe side effects that are caused by unspecific activation of the T cell system. This can result in a potentially lethal CRS, with interleukin 6 (IL-6) apparently playing a key role in the pathophysiology [85,86]. The extent of CRS upon bsAb treatment correlates to overall T cell activation and may be mediated by the following mechanisms:

(i) desired “on-target on-tumor” T cell activation;
(ii) undesired “off-target” effects in the absence of target cells, e.g., due to aggregation, FcR binding, or an overly high affinity of the selected CD3 antibody (Figure 1) [87,88]; and
(iii) undesired “on-target off-tumor” effects occurring upon targeting of target antigens that are not expressed in a highly tumor-restricted manner, as exemplified with Blinatumomab, where the target CD19 is expressed on healthy B cells.

Whereas (i) is inevitable for therapeutic activity, (ii) and (iii) are not associated with efficacy and only limit safely applicable doses, and in turn efficacy. This underlines the importance to choose suitable TAA with highly tumor-restricted expression and to develop suitable bsAb formats that prevent “off-target” effects. With regard to “on-target” toxicities, PSMA, as with most frequently used TAA in PC, is reportedly also expressed—at low levels—in several healthy tissues, and accordingly side effects of T cell stimulation against these tissues have to be considered as potential risk. PSMA-expressing healthy tissues comprise mucosal glands of the eyes and mouth, proximal tubuli of the kidney, mammalian glands, and the gastrointestinal tract [47,48,49,89,90]. However, based on so far available experience, the risk for damage of these tissues seems to be rather limited: Radioactively labeled small molecules that specifically bind to PSMA have been developed and are routinely used for diagnostic imaging as well as targeted radiotherapy. The quite extensive clinical experience with a ^177^Lu-labeled PSMA binding molecule implies that the major side effect is radiation-mediated bone marrow toxicity. In addition, approximately 30% of patients developed xerostomia. Notably, renal or gastrointestinal toxicity has not been reported, suggesting that membrane expression of PSMA on these organs is absent or does not exceed the critical level required for the induction of side effects upon targeted radiotherapy [91].

Another major drawback of some bsAb constructs is the occurrence of anti-drug antibodies (ADAs) [61,92,93], which may not only limit efficacy, but can also cause side effects.

## 6. Current bsAbs under Clinical Evaluation

### 6.1. Pasotuxizumab/BAY 2010112/AMG 212

**Figure 3:**
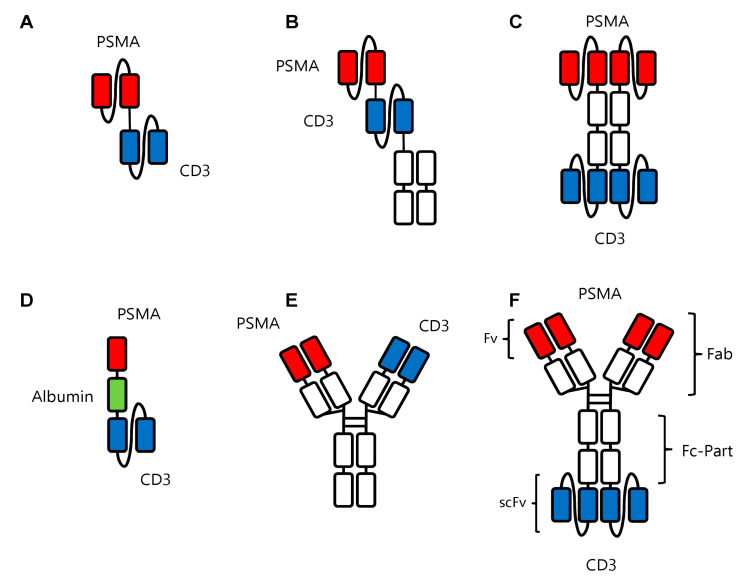
Recombinant bsAb constructs in prostate cancer. (**A**) Pasotuxizumab in the BiTe^TM^ format with the two scFv fragments against PSMA and CD3 shown in red and blue, respectively. (**B**) AMG160 in the HLE BiTE format with the two scFv fragments against PSMA and CD3 shown in red and blue, respectively, and the Fc portion (in white) to increase serum half-life. (**C**) APVO414 with two scFv fragments each directed against PSMA and CD3 in red and blue, respectively, and the Fc portion in white. (**D**) HPN424 in the TriTAC format with a scFv fragment against CD3 in blue and a single-domain antibody against PSMA. Another single-domain antibody (in green) against human serum albumin serves to extend the half-life of the construct. (**E**) JNJ63898081 in the Duobody format with one Fab arm each directed against PSMA and CD3 in red and blue, respectively, and the Fc part in white. (**F**) CC-1 in the IgGsc format containing a modified IgG1 antibody binding to PSMA (red) fused to two scFv fragments (blue) binding to CD3.

The PSMAxCD3 bsAb most advanced in clinical studies is Pasotuxizumab/BAY 2010112/AMG 212, developed by Bayer and Amgen. The molecule, alike the prototypical bsAb Blinatumomab, was constructed in the BiTE format, in which the variable domains of two antibodies are fused together (Figure 3A). Thus, due to this format Pasotuxizumab shares disadvantages of Blinatumomab: The first is the low serum half-life, which is due to the fact that this small molecule does not contain an Fc part [94]. Accordingly, BiTE molecules cannot bind to the FcRn receptor that enables a prolonged serum half-life due to a recycling mechanism induced by this receptor. Low serum half-life requires a cumbersome continuous infusion and comes with higher costs. In a phase I clinical study, Pasotuxizmab was dose-escalated in patients with metastatic CRPC to evaluate maximum tolerated dose (MTD), which was not reached due to premature termination of the trial [61]. The drug was applied as subcutaneous injection (sc) and intravenous (iv) continuous infusion in 31 and 16 patients, respectively. CRS was observed in three patients. Of particular relevance is the observation that all evaluable patients in the sc group developed ADAs, whereas in the iv group, no ADA development was observed. A >50% PSA reduction was reported in nine of 31 and three of 16 patients receiving sc and iv dosing of Pasotuximab, respectively, including two long-term responders [61].

### 6.2. AMG-160

A “next-generation” BiTE molecule is the so-called half-life extended (HLE) BiTE. For this construct, the two scFv fragments of the BiTe are attached to the Fc part of an IgG antibody [95,96], resulting in prolonged half-life (Figure 3B). Besides theoretical and preclinical results, data obtained upon clinical application are available for AMG 160, a PSMAxCD3 HLE-BiTE which was administered via short term iv infusion every 2 weeks in a phase I study. Forty-three patients with metastatic CRPC received ≥1 dose of AMG 160, of which 41 (95.3%) experienced adverse events. However, the maximum tolerated dose was not reached. Overall, 68.6% of patients showed a PSA decline across all dose cohorts and, similar to sc Pasotuximab application [61], 34.3% of patients showed PSA reduction greater than 50%. Despite these promising results, immunogenicity of this construct appears to be a problem: of 30 patients assessed for ADA development, six (20.0%) had developed ADAs at high levels that affect drug exposure, despite the drug being applied iv. An investigation of AMG 160 in combination with pembrolizumab is presently ongoing [96].

### 6.3. APVO414/MOR209/ES414

APVO414/MOR209/ES414 is a PSMAxCD3 bsAb in the so-called ADAPTIR format, which consists of an Fc part as contained in normal IgG antibodies and two scFv fragments for each specificity (Figure 3C). Alike in the HLE-BiTE format, the Fc part prolongs half-life as compared to BiTE constructs [97]. Despite promising preclinical results, the clinical trial was discontinued due to the high immunogenicity of the construct, which is somewhat in line with the findings obtained with the HLE-BiTE: seven of 12 patients with metastatic CRPC receiving once weekly iv infusion of APVO414 developed ADAs [93]. After an amendment to switch to continuous infusion, still 50% of the patients developed ADAs, albeit at lower levels [93]. At present, clinical development of APVO414 has been put on hold [98], probably due to the immunogenicity issue.

### 6.4. HPN424

The so-called Harpoon construct HPN424 is, in contrast to the previously listed constructs, not a bispecific, but rather a trispecific antibody. The construct is generated in the so called TriTAC format (Tri-specific T cell-Activating Construct) and directed against CD3 and PSMA in a monovalent form (Figure 3D). In addition, it contains a specificity binding to human serum albumin (HSA) to prolong serum half-life [99]. The relatively large HSA molecule prevents excretion via the kidney, resulting in a reported half-life of approximately 80 h in cynomolgus monkeys [99,100]. Only the CD3 binding domain of this bsAb is a scFv fragment, whereas the HSA and PSMA specificities are provided by single camelid heavy chain variable domains [101]. This offers the advantage of a higher stability compared to the scFv, but also the potential disadvantage of a higher immunogenicity. Currently, HPN 424 is evaluated in a phase I study to define MTD in advanced CRPC patients. As of May 2020, 44 patients with metastatic CRPC have been treated once weekly. Early signs of clinical activity include PSA reductions in many patients and eight patients being on study for more than 24 weeks. Of note, the data on HPN 424 pharmacokinetics in patients suggest a median half-life of approximately 24.9 h, which is significantly lower than that observed in cynomolgus monkeys. ADAs were observed in approximately 7% of patients [63,102].

### 6.5. JNJ-63898081

The bsAb most closely resembling a physiological IgG antibody is JNJ-63898081 in the so-called duobody format (Figure 3E). This designation refers to bispecific IgG4 antibodies generated by a mechanism that is known as Fab arm exchange [103]. JNJ-63898081 is currently evaluated in a Phase I study (recruiting patients with metastatic CRPC); results have not yet been reported [103].

### 6.6. CC-1

At our institution we have developed a PSMAxCD3 bsAb termed CC-1 which is based on the so-called IgGsc format [104]. In this format, scFv-fragments are covalently attached to the carboxyterminal of the Fc part of an IgG1 antibody (Figure 3F). This offers, on the one hand, the advantage of an extended serum half-life due to its modified Fc-part with its preserved binding to FcRn; on the other hand, it comes with the promise of low immunogenicity. In line with the first notion, pharmacokinetic studies in mice showed the expected long serum half-life as compared to smaller formats missing an Fc part. An open-label, multicenter dose escalation and dose expansion phase I trial is effectively recruiting since 2019 (patients with CRPC) [62], with highly promising clinical results regarding lacking immunogenicity (development of ADAs), safety, and also efficacy.

## 7. Conclusions and Future Perspectives

As of now, convincing therapeutic success of tumor immunotherapy is limited to a minority of patients in case of CI and to hematological diseases as far as bsAbs and CAR-T cells are concerned. In metastatic CRPC, application of sipuleucel-T was reported to prolong OS [21,22,105], but so far plays no major role in daily treatment routine. With regard to bsAbs, the only approved reagent is blinatumomab for treatment of B-ALL. The reasons for the so far limited success of bsAbs (and CAR-T cells) in solid tumors [106,107] are not yet fully understood, but a limited access of immune effector cells to solid tumors appears to constitute a major hurdle [51]. To overcome this limitation, an optimal target antigen should be expressed not only on tumor cells, but also on tumor vessels, allowing for so called dual targeting of both structures, which then results in sufficient influx of immune cells via damaged endothelium and subsequent tumor cell destruction. According to this consideration, PSMA, as employed in most bsAbs under development for treatment of PC, appears to be an ideally suited target antigen. In addition, PSMA displays a highly tumor restricted expression pattern [50,108], which is associated with reduction of both, damage to healthy tissues and undesired “on-target off tumor” T cell activation resulting in CRS. The latter can further be reduced by selecting bsAb formats with diminished aggregation tendency to avoid “off-target” T cell activation, as exemplified by our findings with the IgGsc bsAb format when compared to smaller formats [88,109]. To further prevent the sequelae of CRS, we and others [110] suggest the prophylactic use of the IL6 receptor (IL-6R) antibody tocilizumab to prevent development of CRS in the first place rather than treating CRS once it has arisen. This is based on data that IL-6R blockade, in contrast to dexamethasone, prevents unwanted effects of T cell activation without interfering with therapeutic efficacy [111,112]. It further holds promise to increase the safety of study patients and to allow for application of higher and thus hopefully more effective drug doses.

Another issue with many bsAbs is the often low serum half-life [88,113], which, e.g., in the case of BiTE molecules, required the implementation of cumbersome continuous infusion protocols. Accordingly, several larger, IgG-based molecules like our IgGsc, the duobody format, or half-life extended versions of small constructs (e.g., by fusion to albumin [99] or to PEG derivatives [64]) are presently under development. In this context, the phenomenon of target mediated drug disposition (TMDD) must be considered, which results in accelerated serum elimination particularly at the—compared to mAbs—low doses at which bsAbs usually are applied [113,114]. TMDD is caused by T cells as well as tumor cells acting as “antigen sink”, resulting in a sharp decrease of serum levels of bsAbs at low doses in the range of 0.1 mg/kg [115]. Of note, TMDD is hard to address when using conventional animal models. As an example, half-life of HPN424 was found to be about 80 h in non-human primates, which differs substantially from that reported in humans (approximately 24.9 h) [102]. In addition, half-life is further influenced by the development of ADAs, which at least for some PSMAxCD3 bsAbs occurs in a substantial proportion of treated patients [61]. This underlines the necessity to generate constructs with low immunogenicity.

With regard to achieving optimal efficacy, bsAbs may benefit from combinatorial treatment regimens, especially if one considers that so called “second signals” via co-stimulatory and/or co-inhibitory (“immune checkpoint”) receptors influence the extent and sustainability of T cell responses [8]. One possibility is the combination with CI, which allows for a “mutual benefit”. CI could reinforce the activity of bsAbs by favorably modifying second signals, whereas bsAbs could overcome a putative major hurdle that so far limits efficacy of CI to a small proportion of patients, namely, the lack of sufficient numbers of tumor reactive T cells for amplification by CI. That CI may indeed enhance the activity of bsAbs is suggested by findings that T cell anergy and exhaustion, driven among others by the PD1-PD-L1 axis [116], may affect the efficacy of bsAbs. Blockade of the PD1–PD-L1 axis was reported to reinforce the activity of blinatumomab [117], the CD33 × CD3 BiTE AMG330 [118], and a CD307 × CD3 bsAb evaluated for myeloma treatment [119]. Likewise, the activity of a CD3xCEACAM5xTrop2 construct directed against solid tumors was enhanced when combined with PD1-blockade in an in vivo model [120]. Preliminary results have also been reported for the combination of atezolizumab (anti-PD-L1) with a CEA × CD3 bsAb and for an anti-PD1 antibody combined with a CD20 × CD3 bsAb in colorectal carcinoma and B cell lymphoma, respectively [121,122]. Both studies have described a favorable safety profile of the combination as well as first evidence of activity and clinical responses. In addition, combining CI and bsAbs may lead to induction of epitope spreading, thereby further causing synergistic effects, when tumor reduction by bsAb treatment renders TAA accessible to the immune system and thereby enables induction of specific T cells, which can be boosted by CI. Preclinical data implying epitope spreading have been reported for BiTE bsAbs and CI [123,124,125]. A similar effect may occur when tumor reduction is propelled by CI and the ongoing immune response is boosted by bsAb treatment. The cancer indication—and molecule-spanning synergies—underpin the potential of the combination of CI and bsAbs and have prompted the initiation of clinical studies combining both treatment modalities [121,122,126]. Following the same reasoning, we will shortly initiate a clinical trial evaluating our PSMAxCD3 bsAb CC-1, which contains a PSMA binder with extended reactivity suitable to target squamous cell carcinoma of the lung, in a clinical trial in combination with PD-1 blockade in lung cancer patients (NCT04496674).

Beyond CI, another combinatorial approach involving bsAbs is recently receiving renewed interest: physiologically, stimulation of the TCR/CD3 complex without co-stimulation results in activation-induced cell death and T cell anergy [41]. In line with such observations, as already stated above, it required inclusion of co-stimulatory signaling domains derived from CD28 and/or 4-1BB for CAR-T cells to become therapeutically effective [42]. Already several decades ago we and others have introduced bispecific CD28 antibodies, including in our case the first application to patients [43,44]. However, the clinical development of such BiCos was thereafter impacted largely by the so called “Tegenero incident”, the deleterious cytokine release after application of a—notably monospecific and thus not target cell-restricted—“superagonistic” CD28 antibody [45,46]. That this incident severely compromised the development not only of mono- but also of bispecific CD28 antibodies is somewhat paradoxical, because we consider it the “founding principle” of BiCo construction to avoid undesired “off-target” T cell activation. In fact, BiCos that act in a strictly target-restricted manner offer new perspectives for combinatorial application together with bsAbs that stimulate CD3. Such combinations would (i) prevent T cell anergy and enable induction of an efficient and long-lasting T cells response; (ii) reduce the required dose of CD3-stimulating constructs, with accordingly reduced side effects of CD3-mediated T cell activation; and (iii) profoundly increase tumor specificity by simultaneously targeting two different antigens, resulting in enhanced tumor specificity and reduced toxicity. Thus, within a combinatorial approach, targeted costimulation with BiCos has the potential to improve the specificity and efficacy of nowadays bsAb treatment in a fundamental way.

For both, bsAbs that stimulate CD3 as well as for BiCos that activate CD28, target cell restriction is critical to avoid off-target T cell activation. Regarding CD28 activation, this is particularly difficult to achieve, as—in contrast to most CD3 antibodies—monospecific CD28 antibodies may exert agonistic or even superagonistic activity without binding to target cells or Fc receptors [45,46,127,128]. We have recently developed BiCos directed to different TAAs in our IgGsc format (IgG molecule with two c-terminal single chain moieties) that allow for CD28 costimulation in a completely target cell-restricted manner. Our lead construct is presently undergoing GMP production and, among others, is suitable for combination with our and other PSMAxCD3 constructs.

Notably, Regeneron Pharmaceuticals and Sanofi Aventis as well as investigators at Fred Hutchinson Cancer Research Center have recently reported the development of BiCos for CD28 stimulation and their use in combination with various CD3-stimulating constructs [129,130,131]. However, in one of these cases, a superagonistic antibody has been used for BiCo construction [130] and in another trispecific constructs with targetxCD28xCD3 specificity were generated [131]. Thus, it is at present unclear, and at least questionable, to what extent these constructs meet the critical requirement of target cell-restricted activity.

Taken together, the present developmental activity regarding bsAbs for the treatment of PC is impressive. Novel constructs with favorable properties and combinatorial approaches hold promise to overcome the current limitations, and it can be hoped that in the next few years bsAbs will enable a breakthrough in PC treatment which these patients so highly deserve.

## Data Availability

No new data were created in this study. Data sharing is not applicable to this article.

## Methods

A systematic analysis of the literature was conducted on 1 December 2020 by performing a search on PubMed using the term “prostate cancer” combined with “bispecific”. The search was limited to articles written in English. In addition, a search was conducted using the words “prostate cancer”, “checkpoint”, “bsAb”, and “CAR-T” on clinicaltrials.gov to retrieve data about ongoing studies. Reference lists of key articles were searched for further leads.
